# Supplementary material for: Dopaminergic and opioidergic regulation during anticipation and consumption of social and nonsocial rewards
Source: eLife. 2020 Oct 13;9:e55797. doi: 10.7554/eLife.55797 (PMC7553773; doi:10.7554/eLife.55797)
Supplement: Supplementary file 1. [file elife-55797-supp1.docx]

Description of all elements in a trial

Each trial included (Fig. S1) the following steps: 1) a fixation cross (2 sec), 2) a picture announcing the highest possible reward (high or low, 3 sec), 3) a continuous scale ranging from ‘not at all’ to ‘very much’ to rate (without time limit) wanting of the announced reward (ratings were converted to a 20-point Likert scale), 4) a 2-sec period announcing the effort phase, 5) a 4-sec period of physical effort, during which participants could set the chances of receiving the announced reward by the amount of force they would exert by squeezing the dynamometer with their right hand while receiving visual feedback of the amount of exerted force (sliding average of 1 sec, as percentage of the MVC), 6) a picture announcing the obtained reward (3 sec), which could be high, low, or verylow (the greater participants’ effort, the higher the probability of obtaining the announced reward), 7) a phase of preparation for reward delivery (3 sec for food, 7.3 sec for touch), 8) a phase of reward delivery (2 sec for food, 6.5 sec for touch – this difference in timing was necessary to obtain sufficiently long tactile stimulation, while keeping the overall trial duration similar across reward types), and only in the food trials also instructions to slightly lean back and swallow the obtained reward (duration 3 sec), 9) a relaxation phase (5 sec), and 10) a continuous scale ranging from negative to positive to rate the liking of the obtained reward. In food trials, participants then received water for mouth rinsing, in a way similar to how they had received the food reward. All trials ended with a blank screen for 3 to 4 seconds.

Model tables for explicit measures: wanting, liking and effort

*M****odel tables showing all fixed and random effects.***

|  | **wanting** | | |
| --- | --- | --- | --- |
| *Predictors* | *Estimates* | *CI* | *p* |
| (Intercept) | 3.01 | 2.59 – 3.42 | **<0.001** |
| reward_type_C1 | 0.18 | -0.09 – 0.46 | 0.187 |
| reward_level_C1 | 1.84 | 1.51 – 2.17 | **<0.001** |
| Drug_group_C1 | -0.50 | -1.01 – 0.02 | 0.061 |
| Drug_group_C2 | -0.14 | -0.43 – 0.15 | 0.347 |
| block_new_C1 | -0.16 | -0.21 – -0.12 | **<0.001** |
| reward_type_C1:reward_level_C1 | -0.11 | -0.38 – 0.17 | 0.441 |
| reward_type_C1:Drug_group_C1 | -0.10 | -0.44 – 0.23 | 0.557 |
| reward_type_C1:Drug_group_C2 | 0.07 | -0.12 – 0.26 | 0.499 |
| reward_level_C1:Drug_group_C1 | -0.05 | -0.46 – 0.36 | 0.813 |
| reward_level_C1:Drug_group_C2 | -0.09 | -0.32 – 0.14 | 0.454 |
| reward_type_C1:reward_level_C1:Drug_group_C1 | -0.11 | -0.45 – 0.23 | 0.526 |
| reward_type_C1:reward_level_C1:Drug_group_C2 | -0.04 | -0.23 – 0.16 | 0.701 |
| **Random Effects** | | | |
| σ^2^ | 4.94 | | |
| τ_00_ _sub_ | 5.86 | | |
| τ_11_ _sub.reward_type_C1_ | 2.45 | | |
| τ_11_ _sub.reward_level_C1_ | 3.66 | | |
| τ_11_ _sub.reward_type_C1:reward_level_C1_ | 2.53 | | |
| ρ_01_ | -0.27 | | |
|  | 0.09 | | |
|  | 0.13 | | |
| ICC | 0.75 | | |
| N _sub_ | 131 | | |
| Observations | 10174 | | |
| Marginal R^2^ / Conditional R^2^ | 0.160 / 0.786 | | |

|  | **Effort** | | |
| --- | --- | --- | --- |
| *Predictors* | *Estimates* | *CI* | *p* |
| (Intercept) | 76.25 | 73.49 – 79.00 | **<0.001** |
| reward_type_C1 | -0.02 | -0.99 – 0.96 | 0.972 |
| reward_level_C1 | 4.21 | 3.09 – 5.33 | **<0.001** |
| Drug_group_C1 | -1.44 | -4.84 – 1.96 | 0.407 |
| Drug_group_C2 | 1.02 | -0.91 – 2.95 | 0.304 |
| block_new_C1 | -2.17 | -2.49 – -1.85 | **<0.001** |
| reward_type_C1:reward_level_C1 | 0.13 | -0.83 – 1.09 | 0.789 |
| reward_type_C1:Drug_group_C1 | -0.82 | -2.03 – 0.38 | 0.181 |
| reward_type_C1:Drug_group_C2 | -0.97 | -1.65 – -0.29 | **0.006** |
| reward_level_C1:Drug_group_C1 | 0.36 | -1.02 – 1.74 | 0.607 |
| reward_level_C1:Drug_group_C2 | -0.95 | -1.73 – -0.16 | **0.019** |
| reward_type_C1:reward_level_C1:Drug_group_C1 | -0.56 | -1.75 – 0.62 | 0.354 |
| reward_type_C1:reward_level_C1:Drug_group_C2 | 0.19 | -0.48 – 0.87 | 0.573 |
| **Random Effects** | | | |
| σ^2^ | 213.75 | | |
| τ_00_ _sub_ | 254.79 | | |
| τ_11_ _sub.reward_type_C1_ | 28.85 | | |
| τ_11_ _sub.reward_level_C1_ | 39.09 | | |
| τ_11_ _sub.reward_type_C1:reward_level_C1_ | 28.01 | | |
| ρ_01_ | 0.18 | | |
|  | -0.21 | | |
|  | 0.05 | | |
| ICC | 0.62 | | |
| N _sub_ | 131 | | |
| Observations | 8034 | | |
| Marginal R^2^ / Conditional R^2^ | 0.051 / 0.640 | | |

|  | **liking** | | |
| --- | --- | --- | --- |
| *Predictors* | *Estimates* | *CI* | *p* |
| (Intercept) | 1.72 | 1.37 – 2.07 | **<0.001** |
| reward_type_C1 | 0.17 | -0.04 – 0.37 | 0.120 |
| reward_obtained_ranked_new_C1 | 1.39 | 1.11 – 1.68 | **<0.001** |
| reward_obtained_ranked_new_C2 | 1.53 | 1.34 – 1.72 | **<0.001** |
| Drug_group_C1 | -0.30 | -0.73 – 0.13 | 0.176 |
| Drug_group_C2 | -0.07 | -0.32 – 0.18 | 0.573 |
| block_new_C1 | -0.25 | -0.29 – -0.21 | **<0.001** |
| reward_type_C1:reward_obtained_ranked_new_C1 | -0.10 | -0.35 – 0.15 | 0.429 |
| reward_type_C1:reward_obtained_ranked_new_C2 | -0.04 | -0.19 – 0.11 | 0.575 |
| reward_type_C1:Drug_group_C1 | 0.01 | -0.25 – 0.26 | 0.958 |
| reward_type_C1:Drug_group_C2 | 0.10 | -0.05 – 0.25 | 0.177 |
| reward_obtained_ranked_new_C1:Drug_group_C1 | -0.26 | -0.61 – 0.09 | 0.142 |
| reward_obtained_ranked_new_C2:Drug_group_C1 | -0.06 | -0.29 – 0.18 | 0.646 |
| reward_obtained_ranked_new_C1:Drug_group_C2 | -0.08 | -0.28 – 0.12 | 0.421 |
| reward_obtained_ranked_new_C2:Drug_group_C2 | -0.06 | -0.20 – 0.07 | 0.374 |
| reward_type_C1:reward_obtained_ranked_new_C1:Drug_group_C1 | 0.01 | -0.30 – 0.31 | 0.964 |
| reward_type_C1:reward_obtained_ranked_new_C2:Drug_group_C1 | -0.08 | -0.27 – 0.11 | 0.405 |
| reward_type_C1:reward_obtained_ranked_new_C1:Drug_group_C2 | 0.00 | -0.18 – 0.18 | 0.995 |
| reward_type_C1:reward_obtained_ranked_new_C2:Drug_group_C2 | -0.03 | -0.14 – 0.07 | 0.543 |
| **Random Effects** | | | |
| σ^2^ | 4.75 | | |
| τ_00_ _sub_ | 4.01 | | |
| τ_11_ _sub.reward_type_C1_ | 1.33 | | |
| τ_11_ _sub.reward_obtained_ranked_new_C1_ | 2.44 | | |
| τ_11_ _sub.reward_obtained_ranked_new_C2_ | 1.20 | | |
| τ_11_ _sub.reward_type_C1:reward_obtained_ranked_new_C1_ | 1.84 | | |
| τ_11_ _sub.reward_type_C1:reward_obtained_ranked_new_C2_ | 0.73 | | |
| ρ_01_ | -0.17 | | |
|  | 0.16 | | |
|  | 0.06 | | |
|  | -0.12 | | |
|  | 0.09 | | |
| ICC | 0.72 | | |
| N _sub_ | 131 | | |
| Observations | 10111 | | |
| Marginal R^2^ / Conditional R^2^ | 0.243 / 0.789 | | |

Model tables for implicit measures: facial EMG

| **Pre-Effort Anticipation** | | | | | | | | |
| --- | --- | --- | --- | --- | --- | --- | --- | --- |
|  | **Corrugator Wanting** | | **Corrugator Effort** | | **Zygomaticus Wanting** | | **Zygomaticus Effort** | |
| *Predictors* | *Estimates* | *p* | *Estimates* | *p* | *Estimates* | *p* | *Estimates* | *p* |
| Intercept | 113.03 | **<0.001** | 113.03 | **<0.001** | 131.07 | **<0.001** | 131.01 | **<0.001** |
| Reward Type | -2.89 | **0.001** | -3.03 | **0.001** | -7.88 | **<0.001** | -8.03 | **<0.001** |
| Wanting | -2.80 | **0.006** |  |  | 1.43 | 0.426 |  |  |
| Drug-Naltrexone | 0.36 | 0.730 | 0.49 | 0.734 | -0.14 | 0.964 | -0.05 | 0.987 |
| Drug-Placebo | -1.02 | 0.081 | -1.26 | 0.121 | -2.18 | 0.234 | -2.22 | 0.230 |
| Block | 2.29 | **0.005** | 1.99 | **0.017** | 0.12 | 0.939 | 0.15 | 0.926 |
| Reward Type X Wanting | -0.14 | 0.907 |  |  | -4.16 | **0.010** |  |  |
| Reward Type X Drug-Naltrexone | -0.28 | 0.802 | -0.43 | 0.702 | 2.20 | 0.403 | 2.07 | 0.425 |
| Reward Type X Drug-Placebo | 1.79 | **0.005** | 1.79 | **0.005** | 0.88 | 0.553 | 0.94 | 0.522 |
| Wanting X Drug-Naltrexone | 0.49 | 0.694 |  |  | 2.20 | 0.321 |  |  |
| Wanting X Drug-Placebo | 0.14 | 0.842 |  |  | -0.43 | 0.732 |  |  |
| Reward Type X Wanting X Drug-Naltrexone | -2.02 | 0.158 |  |  | 2.33 | 0.240 |  |  |
| Reward Type X Wanting X Drug-Placebo | -1.50 | 0.068 |  |  | 0.26 | 0.819 |  |  |
| Effort |  |  | -2.60 | **0.012** |  |  | 0.36 | 0.822 |
| Reward Type X Effort |  |  | 0.32 | 0.781 |  |  | -1.65 | 0.296 |
| Effort X Drug-Naltrexone |  |  | 0.41 | 0.746 |  |  | 2.71 | 0.169 |
| Effort X Drug-Placebo |  |  | 0.87 | 0.228 |  |  | -0.62 | 0.579 |
| Reward Type X Effort X Drug-Naltrexone |  |  | -1.15 | 0.420 |  |  | 0.86 | 0.659 |
| Reward Type X Effort X Drug-Placebo |  |  | -0.49 | 0.548 |  |  | 0.99 | 0.373 |
| **Random Effects** | | | | | | | | |
| σ^2^ | 5257.24 | | 5176.21 | | 18167.98 | | 18251.04 | |
| τ_00_ | 0.00 _sub_ | | 84.98 _sub_ | | 579.42 _sub_ | | 587.52 _sub_ | |
| τ_11_ | 17.50 _sub.reward_type1_ | | 21.21 _sub.reward_type1_ | | 277.94 _sub.reward_type1_ | | 264.04 _sub.reward_type1_ | |
|  | 33.70 _sub.z_wanting_ | | 43.19 _sub.z_max_mean_percentage_force_ | | 84.08 _sub.z_wanting_ | | 12.08 _sub.z_max_mean_percentage_force_ | |
|  | 81.93 _sub.reward_type1:z_wanting_ | | 81.85 _sub.reward_type1:z_max_mean_percentage_force_ | |  | |  | |
| ρ_01_ |  | | -0.68 | | -0.53 | | -0.56 | |
|  |  | | -0.64 | | 0.98 | | 0.64 | |
|  |  | | 0.08 | |  | |  | |
| ICC |  | |  | | 0.05 | |  | |
| N | 130 _sub_ | | 130 _sub_ | | 130 _sub_ | | 130 _sub_ | |
| Observations | 7961 | | 7961 | | 7846 | | 7846 | |
| Marginal R^2^/ Conditional R^2^ | 0.007 / NA | | 0.007 / NA | | 0.005 / 0.054 | | 0.005 / NA | |

| **Post-Effort Anticipation** | | | | | | | | |
| --- | --- | --- | --- | --- | --- | --- | --- | --- |
|  | **Corrugator Wanting** | | **Corrugator Effort** | | **Zygomaticus Wanting** | | **Zygomaticus Effort** | |
| *Predictors* | *Estimates* | *p* | *Estimates* | *p* | *Estimates* | *p* | *Estimates* | *p* |
| Intercept | 131.87 | **<0.001** | 132.02 | **<0.001** | 153.12 | **<0.001** | 153.26 | **<0.001** |
| Reward Type | -5.08 | 0.321 | -4.67 | 0.378 | -4.41 | 0.228 | -4.05 | 0.272 |
| Wanting | 3.56 | 0.347 |  |  | 6.82 | **0.010** |  |  |
| Drug-Naltrexone | 2.70 | 0.680 | 2.69 | 0.690 | -1.02 | 0.862 | -0.62 | 0.916 |
| Drug-Placebo | -1.83 | 0.621 | -2.07 | 0.587 | -2.57 | 0.440 | -2.47 | 0.462 |
| Block | -4.42 | 0.203 | -3.73 | 0.289 | 2.29 | 0.260 |  |  |
| Reward Type X Wanting | 2.94 | 0.439 |  |  | 3.00 | 0.231 |  |  |
| Reward Type X Drug-Naltrexone | 4.77 | 0.451 | 4.93 | 0.450 | -0.91 | 0.841 | -0.48 | 0.916 |
| Reward Type X Drug-Placebo | -1.46 | 0.683 | -1.33 | 0.720 | -1.18 | 0.645 | -1.35 | 0.600 |
| Wanting X Drug-Naltrexone | -4.02 | 0.386 |  |  | 0.84 | 0.794 |  |  |
| Wanting X Drug-Placebo | -0.53 | 0.843 |  |  | -0.90 | 0.628 |  |  |
| Reward Type X Wanting X Drug-Naltrexone | 1.51 | 0.746 |  |  | 1.03 | 0.737 |  |  |
| Reward Type X Wanting X Drug-Placebo | -2.22 | 0.408 |  |  | -0.62 | 0.726 |  |  |
| Effort |  |  | 2.96 | 0.424 |  |  | 3.60 | 0.133 |
| Reward Type X Effort |  |  | -1.51 | 0.686 |  |  | -0.29 | 0.894 |
| Effort X Drug-Naltrexone |  |  | -5.39 | 0.233 |  |  | -0.90 | 0.761 |
| Effort X Drug-Placebo |  |  | 2.16 | 0.400 |  |  | -0.85 | 0.615 |
| Reward Type X Effort X Drug-Naltrexone |  |  | -1.27 | 0.782 |  |  | -2.86 | 0.285 |
| Reward Type X Effort X Drug-Placebo |  |  | -0.10 | 0.971 |  |  | -0.14 | 0.929 |
| **Random Effects** | | | | | | | | |
| σ^2^ | 95446.64 | | 95203.19 | | 31936.35 | | 32136.79 | |
| τ_00_ | 2057.60 _sub_ | | 2253.33 _sub_ | | 2369.75 _sub_ | | 2430.81 _sub_ | |
| τ_11_ | 1808.92 _sub.reward_type1_ | | 2036.95 _sub.reward_type1_ | | 1173.05 _sub.reward_type1_ | | 1205.69 _sub.reward_type1_ | |
|  | 105.72 _sub.z_wanting_ | | 84.14 _sub.z_max_mean_percentage_force_ | | 266.22 _sub.z_wanting_ | | 163.73 _sub.z_max_mean_percentage_force_ | |
|  | 116.29 _sub.reward_type1:z_wanting_ | | 140.85 _sub.reward_type1:z_max_mean_percentage_force_ | | 175.11 _sub.reward_type1:z_wanting_ | | 34.38 _sub.reward_type1:z_max_mean_percentage_force_ | |
| ρ_01_ | 1.00 | | 0.97 | | 0.12 | | 0.18 | |
|  | 1.00 | | -0.85 | | 0.71 | | 0.67 | |
|  | 1.00 | | -1.00 | | 0.53 | | -0.13 | |
| N | 130 _sub_ | | 130 _sub_ | | 130 _sub_ | | 130 _sub_ | |
| Observations | 7961 | | 7961 | | 7846 | | 7846 | |
| Marginal R^2^/ Conditional R^2^ | 0.001 / NA | | 0.001 / NA | | 0.003 / NA | | 0.002 / NA | |

| **Delivery** | | | | |
| --- | --- | --- | --- | --- |
|  | **Corrugator Liking** | | **Zygomaticus Liking** | |
| *Predictors* | *Estimates* | *p* | *Estimates* | *p* |
| Intercept | 135.67 | **<0.001** | 195.22 | **<0.001** |
| Reward Type | -18.56 | **0.005** | -70.07 | **<0.001** |
| Liking | -4.18 | 0.122 | -0.52 | 0.801 |
| Drug-Naltrexone | 7.96 | 0.366 | 9.95 | 0.289 |
| Drug-Placebo | -2.30 | 0.644 | -2.25 | 0.671 |
| Block | 0.45 | 0.853 | 0.84 | 0.663 |
| Reward Type X Liking | 0.24 | 0.928 | -3.55 | 0.103 |
| Reward Type X Drug-Naltrexone | -7.66 | 0.341 | -8.66 | 0.379 |
| Reward Type X Drug-Placebo | 2.71 | 0.551 | -2.86 | 0.607 |
| Liking X Drug-Naltrexone | 0.45 | 0.893 | -4.22 | 0.095 |
| Liking X Drug-Placebo | 0.30 | 0.874 | 3.13 | **0.030** |
| Reward Type X Liking X Drug-Naltrexone | 1.76 | 0.587 | -0.54 | 0.838 |
| Reward Type X Liking X Drug-Placebo | -0.12 | 0.948 | 0.75 | 0.622 |
| **Random Effects** | | | | |
| σ^2^ | 58769.95 | | 35686.63 | |
| τ_00_ | 5735.80 _sub_ | | 6924.41 _sub_ | |
| τ_11_ | 4662.24 _sub.reward_type1_ | | 7694.25 _sub.reward_type1_ | |
|  | 101.79 _sub.z_liking_ | | 14.26 _sub.z_liking_ | |
|  | 47.86 _sub.reward_type1:z_liking_ | | 85.27 _sub.reward_type1:z_liking_ | |
| ρ_01_ | -0.88 | | -0.91 | |
|  | 0.68 | | 0.33 | |
|  | -0.56 | | -0.07 | |
| N | 130 _sub_ | | 130 _sub_ | |
| Observations | 9935 | | 9812 | |
| Marginal R^2^/ Conditional R^2^ | 0.008 / NA | | 0.126 / NA | |

| **Relax** | | | | |
| --- | --- | --- | --- | --- |
|  | **Corrugator Liking** | | **Zygomaticus Liking** | |
| *Predictors* | *Estimates* | *p* | *Estimates* | *p* |
| Intercept | 132.08 | **<0.001** | 171.48 | **<0.001** |
| Reward Type | -20.27 | **<0.001** | -39.44 | **<0.001** |
| Liking | -14.41 | **0.001** | 1.07 | 0.594 |
| Drug-Naltrexone | 3.06 | 0.625 | 4.64 | 0.352 |
| Drug-Placebo | -3.56 | 0.317 | -0.82 | 0.770 |
| Block | 4.69 | 0.127 | 5.00 | **0.006** |
| Reward Type X Liking | 8.80 | **0.006** | -0.80 | 0.678 |
| Reward Type X Drug-Naltrexone | 1.19 | 0.831 | -3.46 | 0.396 |
| Reward Type X Drug-Placebo | 3.07 | 0.331 | -3.15 | 0.174 |
| Liking X Drug-Naltrexone | 3.24 | 0.522 | 1.47 | 0.550 |
| Liking X Drug-Placebo | 2.58 | 0.369 | 2.03 | 0.150 |
| Reward Type X Liking X Drug-Naltrexone | -3.71 | 0.344 | -0.70 | 0.769 |
| Reward Type X Liking X Drug-Placebo | -3.21 | 0.151 | -0.74 | 0.584 |
| **Random Effects** | | | | |
| σ^2^ | 92330.01 | | 32436.17 | |
| τ_00_ | 2098.45 _sub_ | | 1665.15 _sub_ | |
| τ_11_ | 1395.31 _sub.reward_type1_ | | 963.08 _sub.reward_type1_ | |
|  | 891.24 _sub.z_liking_ | | 45.97 _sub.z_liking_ | |
| ρ_01_ | -1.00 | | -0.37 | |
|  | -1.00 | | 0.22 | |
| ICC |  | | 0.08 | |
| N | 130 _sub_ | | 130 _sub_ | |
| Observations | 9935 | | 9812 | |
| Marginal R^2^/ Conditional R^2^ | 0.009 / NA | | 0.045 / 0.117 | |

Model tables for Bayesian LMMs:

|  | **Rating of wanting** | |
| --- | --- | --- |
| *Predictors* | *Estimates* | *CI (95%)* |
| Intercept | 2.65 | **1.94 – 3.37** |
| Reward Type | 0.01 | -0.47 – 0.50 |
| Reward Level | 1.87 | **1.32 – 2.45** |
| Amisulpride | 0.99 | -0.03 – 2.07 |
| Naltrexone | 0.06 | -0.94 – 1.09 |
| Block | -0.16 | **-0.21 – -0.12** |
| Reward Type x Reward Level | -0.18 | -0.65 – 0.29 |
| Reward Type x Amisulpride | 0.21 | -0.48 – 0.88 |
| Reward Type x Naltrexone | 0.30 | -0.37 – 0.96 |
| Reward Level x Amisulpride | 0.11 | -0.70 – 0.91 |
| Reward Level x Naltrexone | -0.21 | -1.04 – 0.59 |
| Reward Type x Reward Level x Amisulpride | 0.21 | -0.45 – 0.93 |
| Reward Type x Reward Level x Naltrexone | -0.01 | -0.68 – 0.67 |
| N _sub_ | 131 | |
| Observations | 10174 | |
| Marginal R^2^ / Conditional R^2^ | 0.171 / 0.783 | |

|  | **Rating of liking** | |
| --- | --- | --- |
| *Predictors* | *Estimates* | *CI (95%)* |
| Intercept | 2.24 | **1.53 – 2.93** |
| Reward Type | 0.09 | -0.29 – 0.48 |
| Reward Level | 1.52 | **1.03 – 2.00** |
| Amisulpride | 0.78 | -0.20 – 1.77 |
| Naltrexone | 0.25 | -0.71 – 1.18 |
| Block | -0.31 | **-0.37 – -0.25** |
| Reward Type x Reward Level | -0.13 | -0.51 – 0.27 |
| Reward Type x Amisulpride | -0.10 | -0.64 – 0.46 |
| Reward Type x Naltrexone | 0.10 | -0.44 – 0.64 |
| Reward Level x Amisulpride | -0.15 | -0.83 – 0.55 |
| Reward Level x Naltrexone | -0.12 | -0.80 – 0.56 |
| Reward Type x Reward Level x Amisulpride | 0.30 | -0.26 – 0.85 |
| Reward Type x Reward Level x Naltrexone | -0.01 | -0.57 – 0.55 |
| N _sub_ | 131 | |
| Observations | 10111 | |
| Marginal R^2^ / Conditional R^2^ | 0.110 / 0.575 | |

|  | **Zygomaticus during Delivery** | |
| --- | --- | --- |
| *Predictors* | *Estimates* | *CI (95%)* |
| Intercept | 188.98 | **163.70 – 213.95** |
| Reward Type | -74.22 | **-100.72 – -47.93** |
| Amisulpride | -2.02 | -38.12 – 34.60 |
| Naltrexone | 17.93 | -17.39 – 53.07 |
| Liking | 5.66 | -1.29 – 12.56 |
| Block | 0.85 | -2.95 – 4.68 |
| Reward Type x Liking | -2.14 | -9.34 – 5.16 |
| Reward Type x Amisulpride | 16.56 | -21.21 – 54.19 |
| Reward Type x Naltrexone | -0.55 | -37.37 – 36.68 |
| Amisulpride x Liking | -4.89 | -14.99 – 5.09 |
| Naltrexone x Liking | -13.63 | **-23.37 – -3.68** |
| Reward Type x Amisulpride x Liking | -1.51 | -12.05 – 8.78 |
| Reward Type x Naltrexone x Liking | -2.69 | -12.93 – 7.46 |
| N _sub_ | 130 | |
| Observations | 9812 | |
| Marginal R^2^ / Conditional R^2^ | 0.096 / 0.346 | |
