# Supplementary material for: Dopaminergic and opioidergic regulation during anticipation and consumption of social and nonsocial rewards
Source: eLife. 2020 Oct 13;9:e55797. doi: 10.7554/eLife.55797 (PMC7553773; doi:10.7554/eLife.55797)
Supplement: Transparent reporting form [file elife-55797-transrepform.docx]

***eLife’s* transparent reporting form**

We encourage authors to provide detailed information *within their submission* to facilitate the interpretation and replication of experiments. Authors can upload supporting documentation to indicate the use of appropriate reporting guidelines for health-related research (see [EQUATOR Network](http://www.equator-network.org/%20)), life science research (see the [BioSharing Information Resource](https://biosharing.org/)), or the [ARRIVE guidelines](http://www.plosbiology.org/article/info:doi/10.1371/journal.pbio.1000412) for reporting work involving animal research. Where applicable, authors should refer to any relevant reporting standards documents in this form.

If you have any questions, please consult our Journal Policies and/or contact us: [editorial@elifesciences.org](mailto:editorial@elifesciences.org).

**Sample-size estimation**

- You should state whether an appropriate sample size was computed when the study was being designed
- You should state the statistical method of sample size computation and any required assumptions
- If no explicit power analysis was used, you should describe how you decided what sample (replicate) size (number) to use

Please outline where this information can be found within the submission (e.g., sections or figure legends), or explain why this information doesn’t apply to your submission:

Sample size was decided based on recent previous work using the same compounds and doses in a 3-group between-subjects design (see lines 400-401 in the manuscript).

Group size in Weber et al. (2016) was 41 (amisulpride), 40 (naltrexone), and 40 (placebo). We therefore aimed for a minimum of 40 participants per group.

Our final group sizes were 42, 44, and 45 respectively for amisulpride, naltrexone, and placebo (see lines 117, 118 in the manuscript).

**Replicates**

- You should report how often each experiment was performed
- You should include a definition of biological versus technical replication
- The data obtained should be provided and sufficient information should be provided to indicate the number of independent biological and/or technical replicates
- If you encountered any outliers, you should describe how these were handled
- Criteria for exclusion/inclusion of data should be clearly stated
- High-throughput sequence data should be uploaded before submission, with a private link for reviewers provided (these are available from both GEO and ArrayExpress)

Please outline where this information can be found within the submission (e.g., sections or figure legends), or explain why this information doesn’t apply to your submission:

This experiment was performed once per participant.

All the data (single trials in long format) and scripts to perform statistical analyses and graphical representations (to be run in the software R) are available on open science framework (OSF). The link (https://bit.ly/35UtUvw) is provided at line 666 of the manuscript. The data are available as tab-delimited .txt files, which can be opened in Excel or other programs. Most variable names are self-explanatory, others are indicated in the scripts.

On lines 680 of the manuscript we describe how outliers were identified in the behavioral data: “Behavioral data were analyzed in the following manner. Outlier trials were defined as those with a rating of wanting, rating of liking, or amount of exerted force, which was greater/smaller than the subject’s mean +/- 2 times the subject’s standard deviation. This led to an average rejection of 6.56 trials per participant (SD = 3.71). The total number of excluded trials did not differ significantly between groups (*t*(133) = -1.28, *p* = .20).”

On lines 704 of the manuscript we describe how outliers were identified in the EMG data: “We excluded for each participant trials on which the average amplitude in the baseline period (one second during fixation) of the corrugator or zygomaticus muscles was lower than M−2*SD, or higher than M+2*SD (M = average amplitude over all trials' baselines for the respective muscle and participant). On average, this led to the rejection of 7.7 % of trials per participant (*SD* = 2.5).”

**Statistical reporting**

- Statistical analysis methods should be described and justified
- Raw data should be presented in figures whenever informative to do so (typically when N per group is less than 10)
- For each experiment, you should identify the statistical tests used, exact values of N, definitions of center, methods of multiple test correction, and dispersion and precision measures (e.g., mean, median, SD, SEM, confidence intervals; and, for the major substantive results, a measure of effect size (e.g., Pearson's r, Cohen's d)
- Report exact p-values wherever possible alongside the summary statistics and 95% confidence intervals. These should be reported for all key questions and not only when the p-value is less than 0.05.

Please outline where this information can be found within the submission (e.g., sections or figure legends), or explain why this information doesn’t apply to your submission:

We describe analyses on line 666: “Group comparisons for age, BMI, MVC, PANAS scores, and side effects, were made with linear regressions using the lm() function. Differences in ranking of rewards across drug groups were tested with separate ordinal regressions by Reward Type (food, touch), using the package *ordinal*.

All other analyses were done with linear mixed effects models (LMMs), fitted through restricted maximum likelihood (REML) estimation, using the lmer() function of the *lmerTest* package in R (which adds p values to the lme4 output; Bates et al., 2014; R Core Team, 2019), and with helmert contrast coding. In comparison to ANOVAs, LMMs reduce Type-I errors and allow for the better generalization of findings (Judd et al., 2012). To control for the effect of time – possibly inducing fatigue and/or habituation (Fig. S3) – the four blocks were recoded to two blocks by Reward Type, and entered as covariates to the LMMs. Figures (except 1 and S1) were created in R using the packages *ggplot2, ggpirate, and cowplot*.”

And on line 725: “We controlled for the false discovery rate (FDR) associated with multiple testing of the EMG data using the Benjamini-Hochberg method (Benjamini & Hochberg, 1995). Model tables with un-corrected p-values can be found in the Supporting Information.”

Effect sizes for main and interaction effects (similar to partial eta squared in ANOVAs) are currently not available for LMMs.

(For large datasets, or papers with a very large number of statistical tests, you may upload a single table file with tests, Ns, etc., with reference to sections in the manuscript.)

**Group allocation**

- Indicate how samples were allocated into experimental groups (in the case of clinical studies, please specify allocation to treatment method); if randomization was used, please also state if restricted randomization was applied
- Indicate if masking was used during group allocation, data collection and/or data analysis

Please outline where this information can be found within the submission (e.g., sections or figure legends), or explain why this information doesn’t apply to your submission:

Allocation of participants to the groups was random and decided before the start of the experiment. Concretely, each participant number was associated beforehand with one of the 3 groups. This was done separately for male (even numbers) and female (odd numbers) participants.

**Additional data files (“source data”)**

- We encourage you to upload relevant additional data files, such as numerical data that are represented as a graph in a figure, or as a summary table
- Where provided, these should be in the most useful format, and they can be uploaded as “Source data” files linked to a main figure or table
- Include model definition files including the full list of parameters used
- Include code used for data analysis (e.g., R, MatLab)
- Avoid stating that data files are “available upon request”

Please indicate the figures or tables for which source data files have been provided:

All the data (single trials in long format) and scripts to perform statistical analyses and graphical representations (to be run in the software R) are available on open science framework (OSF). The link (https://bit.ly/35UtUvw) is provided at line 666 of the manuscript. The data are available as tab-delimited .txt files, which can be opened in Excel or other programs. Most variable names are self-explanatory, others are indicated in the scripts.
